# Effect of body mass index on post-treatment oral function in patients with oral cancer: a cross-sectional study

**DOI:** 10.1038/s41598-024-67246-9

**Published:** 2024-09-19

**Authors:** Yukiho Shimamura, Yuhei Matsuda, Mayu Takeda, Reon Morioka, Tatsuhito Kotani, Takahiro Kanno

**Affiliations:** Department of Oral and Maxillofacial Surgery, Shimane University Faculty of Medicine, 89-1, Enyacho, Izumo, Shimane 693-8501 Japan

**Keywords:** Body mass index, Oral cancer, Oral function, Postoperative oral dysfunction, Tongue pressure, Oral cancer, Surgical oncology

## Abstract

This single-center cross-sectional study used sequential sampling to examine the influence of body mass index (BMI) on oral function after oral cancer treatment. Patients who completed primary oral cancer treatment between September 2019 and March 2023 (102 patients, 74 male [72.5%] and 28 female [27.5%]; mean age, 69.6 years) were analyzed. Patient background data were collected from electronic medical records. Post-treatment oral function measurements were conducted on all patients using six assessment tools. Statistical analysis was conducted using Pearson’s correlation coefficient, one-way analysis of variance, the Jonckheere–Terpstra test, and multiple linear regression. Pre-treatment BMI showed a statistically significant relationship with postoperative oral function, particularly tongue pressure (*P* = 0.01). While the mean values of the groups showed no significant differences, the Jonckheere–Terpstra test revealed a statistically significant trend toward a stepwise increase in tongue pressure for each BMI group (*P* = 0.03). Multiple linear regression analysis revealed a statistically significant correlation between tongue pressure and pre-treatment BMI (*P* < 0.05). Pre-treatment BMI was significantly associated with tongue pressure. Since BMI is a variable factor that can be controlled by nutritional therapy even before treatment, nutritional intervention, weight control, and treatment strategies including reconstructive interventions to maintain tongue pressure may be important in oral cancer treatment.

## Introduction

Globally, males face a higher risk of oral cancer, with over 400,000 new cases and an estimated 200,000 deaths annually^1^. Despite the development of novel anticancer therapies such as immune checkpoint inhibitors, surgical resection remains the first choice for oral cancer treatment, with radiation therapy and chemotherapy positioned as postoperative adjuvant therapies^2^. Following oral cancer surgery, functional impairment of the primary site, hoarseness due to neck dissection, and motor impairment of the upper extremities occur, and diverse adverse events occur when radiotherapy and chemotherapy are included^3^. In particular, oral cancer treatment causes functional as well as esthetic impairment, with reportedly lower quality of life (QoL) of patients with oral cancer than that of patients with other cancers^4^.

In recent years, various parameters, such as oral hygiene, oral dryness, occlusal force, tongue-lip motor function, tongue pressure, masticatory function, and swallowing function, have been commonly used to measure oral function in the field of gerodontology^5^. In the Japanese healthcare system, these oral function measurements function as diagnostic tools for oral hypofunction and are validated as assessment indicators^5^. In healthy older populations, oral hypofunction or poor oral function not only contributes to frailty and sarcopenia but also affects survival rate^6^. Matsuda et al. reported that these oral function measurements designed for healthy older patients are also applicable to patients with oral cancer, and they defined the rapid decline in oral function after oral cancer treatment as postoperative oral dysfunction^7^. Studies on healthy older populations have reported background factors that affect oral function, including body mass index (BMI); however, these factors remain unknown in patients with oral cancer^8^.

Numerous studies have reported the effects of BMI on cancer treatment outcomes. Low BMI has been reported to decrease response rates to treatment and survival rates in patients receiving lung cancer treatment^9^ and decrease the survival rate of patients receiving liver cancer treatment^10^. In contrast, high BMI increases complication rates in the treatment of colorectal and esophageal cancers^11,12^. Hence, too high or too low BMI may be a factor influencing cancer treatment outcomes.

The influence of BMI on oral cancer treatment has been reported previously. Low BMI (< 18.5 kg/m^2^) has been identified as an independent factor, along with low albumin levels and low prognostic nutritional index, for reducing overall survival after treatment in patients with oral cancer^13^. Sarcopenia, a loss of muscle mass that is strongly associated with BMI, has also been reported as a risk factor for complications and poor survival rates in oral cancer treatment^14,15^. Additionally, sarcopenia is associated with high complication rates during cancer treatment, increased adverse event rates in chemotherapy, and lower success rates in reconstructive surgery^16,17^.

Nonetheless, a meta-analysis examining the relevance of BMI in the treatment of head and neck cancers, including oral cancer, revealed some paradoxes. According to the meta-analysis, overweight (BMI: 25–30 kg/m^2^) or underweight (BMI: < 18.5 kg/m^2^) shows decreased survival rate compared with normal weight (BMI: 18.5–25 kg/m^2^), while the risk of decreased survival rate loses its significance when BMI exceeds 30 kg/m^2^^18^. This suggests the presence of other unknown factors in the association between BMI and survival rate. Most previous studies have reported on oral cancer survival rate and BMI. In healthy adults, higher oral function is reported to be associated with higher BMI^19^, and in patients with gastric cancer, oral function declines with a decrease in BMI^20^. However, no reports have examined the association between comprehensive oral function and BMI in patients with oral cancer. Therefore, we hypothesized that the higher the oral function after oral cancer treatment, the higher the BMI. In addition, survival is not necessarily the primary treatment outcome for older patients with oral cancer in a super-aged society such as Japan, and oral function assessment and QoL after oral cancer treatment are crucial. Therefore, this study aimed to examine the impact of BMI on oral function after oral cancer treatment and its relevance.

## Methods

### Patient eligibility

This was a single-center cross-sectional study that used a sequential sampling method. Data from patients who completed primary oral cancer treatment between September 2019 and March 2023 were used. Patient eligibility criteria were as follows: diagnosis with primary oral squamous cell carcinoma; treatment at the Shimane University Hospital Oral and Maxillofacial Surgery/Oral Care Center followed by standard treatment according to the National Comprehensive Cancer Network version 8.0 guidelines; adults aged ≥ 20 years; and ability to complete the questionnaire. Exclusion criteria were as follows: cases in which oral function could not be measured due to death or cognitive function decline and cases of optional or nonstandard treatment based on the guidelines. The study was approved by the Medical Research Ethics Committee of Shimane University Faculty of Medicine (number 4041). Written informed consent was obtained from each participant before participation in the study.

### Patient background data

The following patient background data were collected from electronic medical records: sex (male/female), age (years), primary tumor site (tongue, gingiva, palate, oral floor, and buccal mucosa), clinical cancer stage, treatment method (surgery, surgery and radiotherapy, and surgery and chemoradiotherapy), presence of neck dissection, and presence of reconstructive surgery.

### Body mass index

BMI (kg/m^2^) was measured at the time of hospitalization prior to treatment; data were collected before completion of the primary treatment and patient’s return to society. To determine the change in BMI, pre-treatment data were subtracted from post-treatment data, and to obtain percentiles, pre-treatment data were divided by post-treatment data. BMI was divided into low (< 18.5 kg/m^2^), normal (18.5–23.0 kg/m^2^), and high groups (> 23.0 kg/m^2^) based on the World Health Organization (WHO) criteria^18^.

### Oral function measurement

For oral function measurement, data were collected on six of the seven items recommended by the Japanese Society of Gerodontology for diagnosis of postoperative oral dysfunction: number of microorganisms, oral dryness, occlusal force, tongue pressure, masticatory function, and swallowing function^5^. The number of microorganisms was measured by collecting samples from the center of the tongue dorsum using a rapid oral detection apparatus (Bacterial counter; Panasonic Healthcare, Tokyo, Japan). Oral dryness was measured using an oral moisture checker (Mucus; Life, Saitama, Japan), and the median of three measurements on the dorsum of the tongue was recorded. The occlusal force was measured using a pressure-sensitive paper (Dental Prescale Occluzer; GC, Tokyo, Japan) by clenching for 3 s at the intercuspal position. If the individual had a denture, the occlusal force was measured with the denture in place. Tongue pressure was measured at the center of the dorsum of the tongue using a tongue pressure measuring instrument (TPM-01; JMS, Hiroshima, Japan). Masticatory function was measured using a masticatory ability testing system (Gluco Sensor GS-II; GC, Tokyo, Japan), whereas swallowing function was assessed using a 10-item questionnaire (EAT-10) with a 5-point Likert scale (0 = no problem; 4 = severe problem) developed by Belafsky in 2008. The EAT-10 has a maximum total score of 40, with higher scores indicating poor swallowing function as proposed by Matsuda et al.^7^. All oral function measurements were taken just prior to the patient's return to society after primary treatment according to standard treatment of the National Comprehensive Cancer Network version 8.0 guidelines. Measurements were taken with dentures and palatal augmentation prosthesis in place whenever possible.

### Statistical analysis

The Shapiro–Wilk test was used to confirm normality of the data. Continuous data are described as mean and standard deviation, and categorical data as number and percentages. Pearson’s correlation coefficient was calculated to determine the association between continuous data. The three BMI groups were analyzed using one-way analysis of variance and the Jonckheere–Terpstra test. Multiple linear regression analysis (forced entry method) was conducted as a multivariate analysis, considering confounding factors. Statistical analyses were performed using SPSS version 27 (SPSS Japan Inc., Tokyo, Japan). Two-tailed p-values were calculated for all analyses, and the alpha level of significance was set at *P* < 0.05.

## Results

### Demographic data and clinical characteristics

This survey included 102 consecutive patients who underwent treatment for primary oral cancer; among them, 74 (72.5%) were male and 28 (27.5%) were female. The mean age was 69.6 years (standard deviation 13.6). The mean pre-treatment BMI was 21.5 kg/m^2^ (standard deviation 4.0), and the mean rate of change after treatment was -8.1% (7.2). The primary tumor sites were tongue and gingiva in 86 patients (84.3%); 64 patients (62.7%) had advanced cancer at clinical stages III and IV. Detailed information is provided in Table 1.

**Table 1:** Demographic and clinical characteristics (N = 102).

Variables	Categories	N (%), mean [SD]
Sex	Male	74 (72.5)
	Female	28 (27.5)
Age (years)		69.6 [13.6]
Body mass index (kg/m^2^)		21.5 [4.0]
Change of body mass index (%)		− 8.1 [7.2]
Primary tumor sites	Tongue	45 (44.1)
	Gingiva	41 (40.2)
	Palate	3 (2.9)
	Oral floor	5 (4.9)
	Buccal mucosa	8 (7.8)
Clinical cancer stage	I	26 (25.5)
	II	12 (11.8)
	III	14 (13.7)
	IV	50 (49.0)
Treatment method	Surgery	57 (55.9)
	Surgery and radiotherapy	18 (17.6)
	Surgery and chemoradiotherapy	27 (26.5)
Neck dissection	Yes	64 (62.7)
Reconstructive surgery	Yes	58 (56.9)
Oral function measurement	Microorganisms (Grade)	3.3 (1.5)
	Oral dryness	23.2 (5.1)
	Occlusal force (N)	313.0 (333.3)
	Tongue pressure (kPa)	16.4 (11.6)
	Masticatory function (mg/dL)	97.1 (77.5)
	EAT-10	14.7 (11.4)

*SD* standard deviation, EAT-10, Eating Assessment Tool-10.

### Relationship between BMI and postoperative oral function

**Figure 1:**
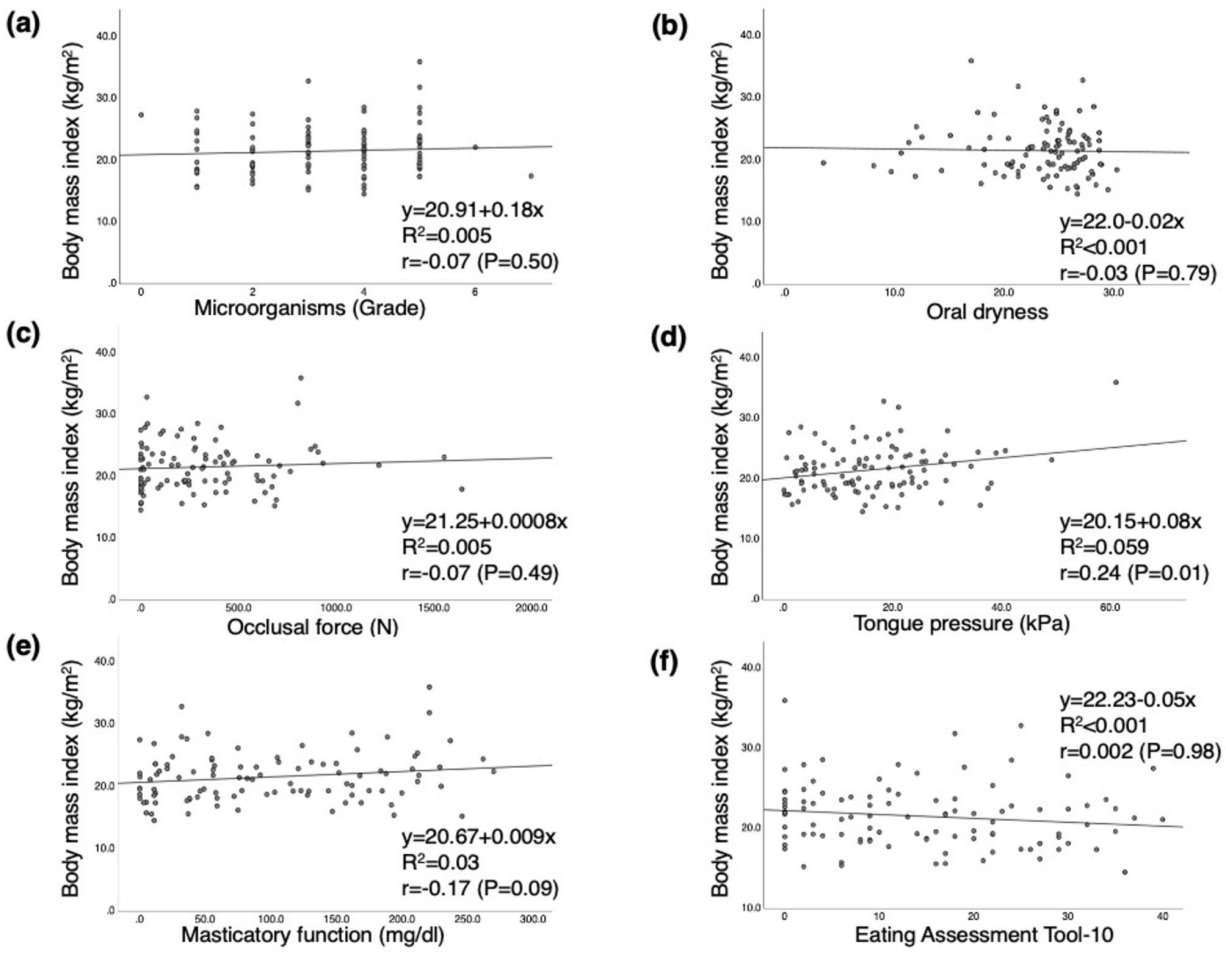
Relationship between pre-treatment body mass index and post-treatment oral function. (**a**) Microorganisms, (**b**) Oral dryness, (**c**) Occlusal force, (**d**) Tongue pressure, (**e**) Masticatory function, (**f**) Eating Assessment Tool-10.

**Figure 2:**
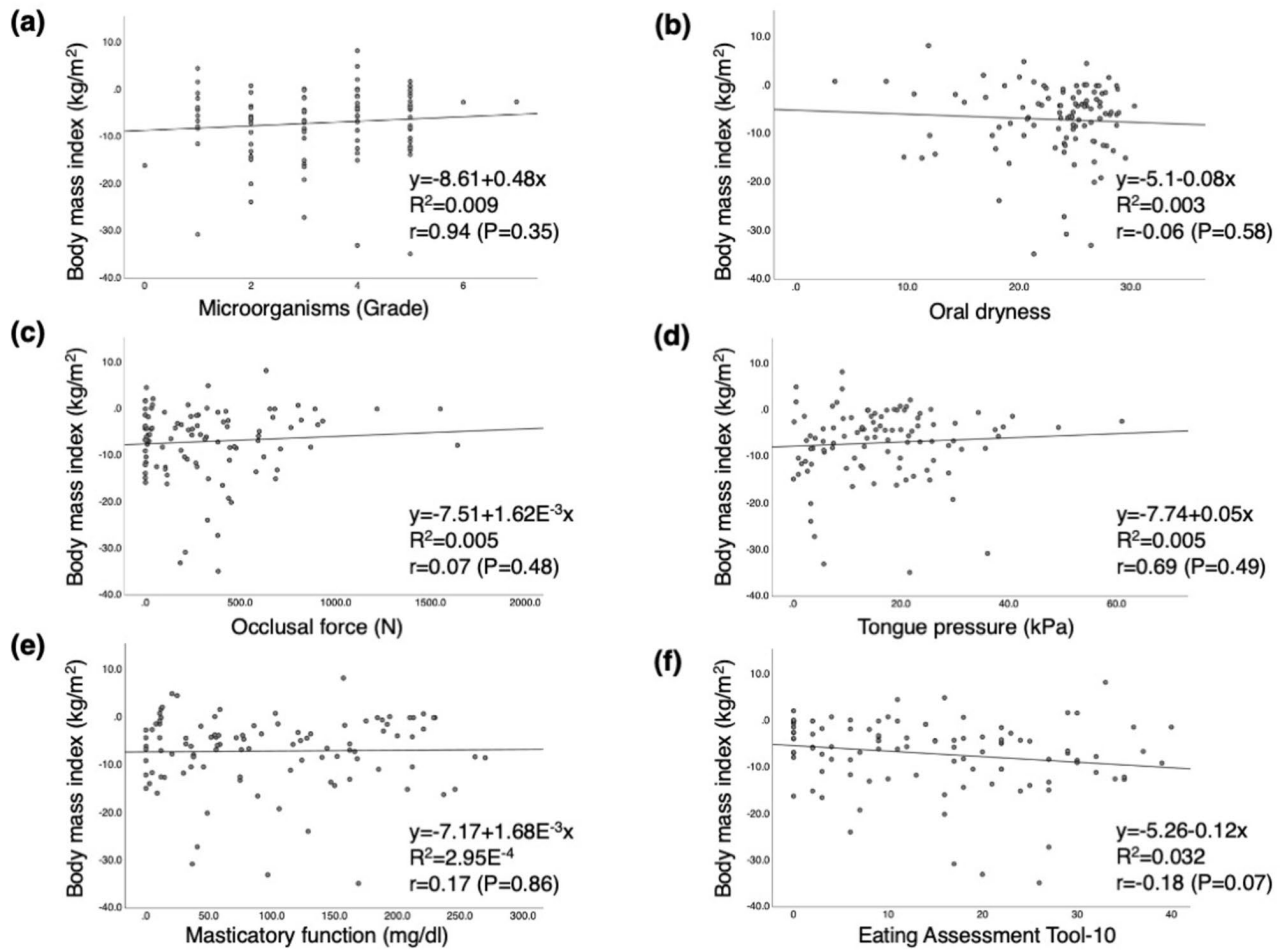
Rate of change of body mass index before and after treatment and its relation to oral function after treatment, (**a**) Microorganisms, (**b**) Oral dryness, (**c**) Occlusal force, (**d**) Tongue pressure, (**e**) Masticatory function, (**f**) Eating Assessment Tool-10.

Pre-treatment BMI showed a significant relationship with postoperative oral function, especially tongue pressure (r = 0.24, *P* = 0.01), as shown in Fig. 1. Figure 2 depicts the relationship between the rate of change in BMI after treatment and oral function. No significant associations were observed with postoperative oral function.

### Group comparison of pre-treatment BMI based on WHO criteria

**Figure 3:**
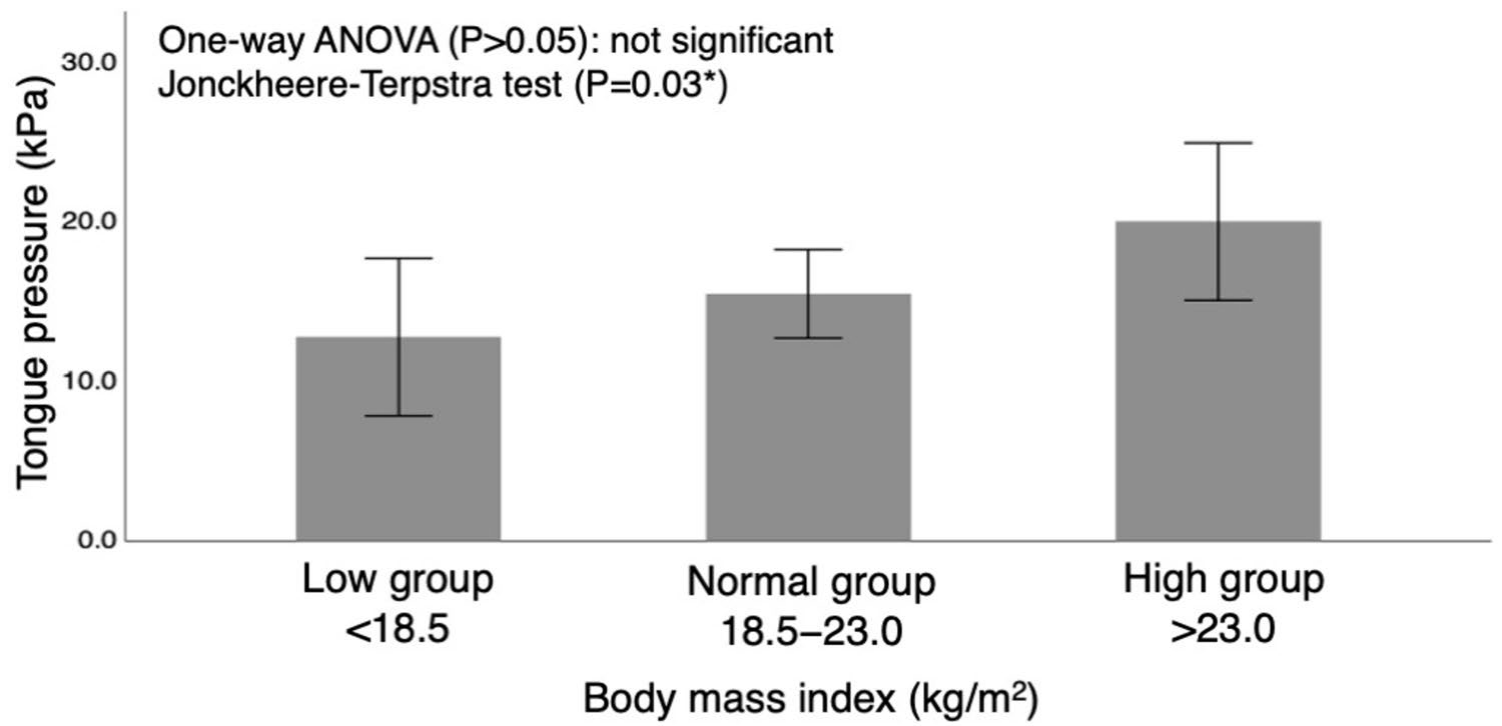
Group comparison of pre-treatment body mass index based on the World Health Organization (WHO) criteria. ANOVA, analysis of variance.

Although there were no significant differences in the mean values among the groups, the Jonckheere–Terpstra test showed a significant trend toward a stepwise increase in tongue pressure in each BMI group (*P* = 0.03; Fig. 3).

### Multivariate analysis

The following items were found to be associated with tongue pressure (Table 2): gingiva as a primary tumor site (β = 9.01 [95% confidence interval: 4.22–13.79], *P* = 0.001), others as a primary tumor site (β = 6.90 [95% confidence interval: 0.44–13.36], *P* = 0.04), clinical cancer stage (β =  − 2.99 [− 6.00– − 0.02] *P* = 0.05), neck dissection (β = 9.38 [95% confidence interval: 1.81–16.95] *P* = 0.02), reconstructive surgery (β =  − 6.51 [95% confidence interval: − 11.49– − 1.54], *P* = 0.01), and BMI (β = 0.74 [95% confidence interval: 0.16–1.32] *P* = 0.01).

**Table 2:** Relationship between tongue pressure and pre-treatment body mass index.

Variables	β (95% confidence interval)	Standardized β	*P*-value
Age	0.58(− 0.11–0.23)	0.07	0.49
Sex	0.28(− 4.40–4.96)	0.01	0.91
Primary tumor site (Gingiva)	9.01(4.22–13.79)	0.38	0.001*
Primary tumor site (Others)	6.90(0.44–13.36)	0.22	0.04*
Clinical cancer stage	− 2.99(− 6.00– − 0.02)	− 0.33	0.05*
Surgery	− 0.29(− 13.52–12.94)	− 0.01	0.97
Surgery and radiotherapy	− 2.34(− 13.70–9.02)	− 0.10	0.68
Surgery and chemoradiotherapy	− 1.64 (− 8.32–5.05)	− 0.07	0.63
Neck dissection	9.38 (1.81–16.95)	0.39	0.02*
Reconstructive surgery	− 6.51(− 11.49– − 1.54)	− 0.28	0.01*
Body mass index	0.74 (0.16–1.32)	0.25	0.01*

*Significant difference.

## Discussion

The main finding of this study was the association between pre-treatment BMI and postoperative tongue pressure. The components contributing to tongue pressure include the intrinsic tongue muscles (transverse and longitudinal muscles), extrinsic tongue muscles (genioglossus, hyoglossus, styloglossus, and palatoglossus muscles), nerves (hypoglossal nerve), tongue volume, and palate morphology^21,22^. Surgical resection of muscles and nerves leads to a decreased tongue volume and range of motion, resulting in decreased tongue pressure^23^. Radiation therapy and chemotherapy also contribute to functional decline, although to a smaller magnitude than organic morphological changes caused by tongue resection^24^. Reconstructive surgery has long been used as a countermeasure against the development of organic defects in the tongue^25^. It has also shown esthetic and functional benefits, with many reports of tongue function restoration due to improvements in surgical equipment and techniques^26^. In addition to treatment-related factors, individual patient factors also influence tongue pressure. Treatment with palatal augmentation prostheses is effective because the volume occupied by the tongue in relation to the oral cavity is important for increasing tongue pressure^27^. During swallowing, the volume of the tongue occupying the oral cavity and palate height are important factors^28^. Since the analysis in this study was adjusted for the effects of treatment-related factors such as surgical site, reconstructive surgery, and neck dissection, individual patient factors may have an independent effect on postoperative tongue pressure. It is important to note that while the palatal height is a constant patient factor, BMI is a variable patient factor that can be addressed.

Although this study revealed the relationship between pre-treatment BMI and post-treatment tongue pressure, the results are consistent with and can be explained by existing literature. First, tongue thickness and BMI have been reported to be positively correlated^29^. In particular, since the posterior part of the tongue has more adipose tissue than the anterior part, having a higher BMI could be advantageous in increasing tongue pressure^30^. Furthermore, since tongue thickness has been reported to be directly related to tongue pressure, it is reasonable to assume that a higher BMI will produce a higher tongue pressure after oral cancer treatment^31^. Patients with oral cancer already have poor oral function and nutritional status before treatment^32^. In particular, a strong association between BMI and sarcopenia has been reported, and older patients with oral cancer may require treatment when sarcopenia and low BMI coexist^33^. Sarcopenia is another factor that contributes to decreased tongue pressure^34^. Therefore, attention should be paid to the decrease in post-treatment tongue pressure in patients falling into the low BMI category according to the WHO criteria. Moreover, enhanced nutritional therapy is advisable before treatment initiation^35^.

A minor finding of this study was that the weight loss resulting from treatment was insufficient to significantly impact tongue pressure or other oral functions. Patients in this study received the nutritional management necessary for general oral cancer treatment and the necessary support from a multidisciplinary team, including a dietitian, physical therapist, and speech-language pathologist. In a randomized controlled trial examining the effects of nutritional therapy on oral cancer treatment, Hannah et al. reported a decrease in BMI of approximately 1–2 kg/m^2^ at treatment completion in the group receiving nutritional intervention^36^. In our patients, the BMI decrease rate was − 8.1% (approximately 1.7 in terms of BMI), indicating effective nutritional management during treatment. Therefore, these results suggest that with appropriate nutritional management, weight loss associated with oral cancer treatment is unlikely to affect postoperative oral function. However, these results also suggest that early nutritional treatment, even before surgery, is more important.

Prior to the surgery, it is crucial to implement measures to prevent the expected decrease in tongue pressure. The tongue and palate are the primary tumor sites expected to impact tongue pressure. In many cases, both pedicled flaps (such as pectoralis major myocutaneous flap and submental island flap) and free flaps (forearm flap, abdominis musculocutaneous flap, and anterolateral thigh flap) are options for reconstructive surgery; however, it should be considered that both types of skin flaps undergo medium- to long-term shrinkage, rendering the preservation of morphology uncertain^37^. In contrast, restoration of tongue pressure through dental prosthetic treatment (palatal augmentation prosthesis) is recommended because the device can be modified as often as needed, with a relatively strong evidence supporting its efficacy^24^. However, increasing tongue pressure alone is inadequate; a comprehensive oral cancer treatment strategy that considers the transport type of postoperative oral dysfunction (Matsuda–Kanno classfication) is needed^7^. For this purpose, collaboration among oral surgeons, dentists, dental hygienists, and dental technicians is considered crucial.

This study has some limitations. First, data on the extent of primary site resection or nutritional indices other than BMI were not collected or considered in the analysis. Nevertheless, this remains the first study to highlight that BMI, a variable factor that can be easily addressed before treatment, is associated with post-treatment oral function, a significant postoperative outcome. Second, being a cross-sectional study, variations in function over time were not considered. Third, differences between facilities were not considered, as this was a single-center study. In particular, inter-institutional differences may be greater in oral function management than in oral cancer treatment methods. Therefore, additional multicenter studies are necessary to confirm our present findings. The results of the present study are clinically significant, as they suggest a possible need for greater flap volume in reconstructive surgery to maintain and improve BMI and tongue pressure after oral cancer treatment. In addition, the potential effectiveness of dental prosthodontic treatment and palatal augmentation prosthesis in improving tongue pressure has been demonstrated. Future interventional studies are needed to determine the effect of increased BMI as a result of enhanced nutritional therapy prior to oral cancer treatment on postoperative tongue pressure.

## Conclusion

Pre-treatment BMI was significantly associated with post-treatment tongue pressure. As BMI is a variable factor that can be controlled by nutritional therapy even before treatment, the results suggest that early nutritional intervention and weight control may be important in oral cancer treatment.

## Data availability

The datasets used and/or analyzed during the current study are available from the corresponding author on reasonable request.
